# Height, VKORC1 1173, and CYP2C9 Genotypes Determine Warfarin Dose for Pediatric Patients with Kawasaki Disease in Southwest China

**DOI:** 10.1007/s00246-018-1957-x

**Published:** 2018-08-18

**Authors:** Dan Yang, Hongyu Kuang, Yuanlin Zhou, Chunqiong Cai, Tiewei Lu

**Affiliations:** 1 Department of Cardiology, Children’s Hospital of Chongqing Medical University; Ministry of Education Key Laboratory of Child Development and Disorders, 136# Zhongshan 2nd Rd. Yuzhong District, Chongqing, 400014 China; 2 China International Science and Technology Cooperation Base of Child Development and Critical Disorders, Chongqing Key Laboratory of Pediatrics, Chongqing, 400014 China; 3 Pediatric Department, People’s Hospital of Qijiang District, Chongqing, Chongqing, 400014 China

**Keywords:** Warfarin, Kawasaki disease, Height, Gene

## Abstract

Long-term oral warfarin is recommended in pediatric Kawasaki disease patients with large coronary artery aneurysms; however, heterogeneity is considerable. This study aimed to determine variables affecting warfarin dosage in Kawasaki disease. The enrolled individuals (194 children) were divided into four groups: (1) Cases with severe coronary artery lesions (CAL) of IV to V degrees or thrombogenesis treated with oral warfarin were assigned to Group A; (2) Group B, CAL of I degrees; (3) Group C, CAL of II and III degrees cases with small or medium-sized CAL not treated with warfarin; (4) Group D, normal children without Kawasaki disease. The relevant genotypes of CYP2C9, VKORC1 (1173, − 1639, and 3730), and CYP4F2 were assessed. There were no statistically significant differences in CYP2C9, VKORC1, and CYP4F2 mutation frequencies among the 4 groups. In the 44 Group A patients, demographic features, clinical characteristics, and genotypes were recorded, and their associations with warfarin dose variability were assessed. Multivariate linear regression analysis revealed that height, VKORC1 1173, and CYP2C9 accounted for 61.2%, 7.9%, and 4.3% of dosing variability, respectively. Conclusions: Patient height is the main factor determining warfarin dosage, while genotype effects on warfarin dosage vary among studies. New formula should be defined using data obtained from children in cases with demonstrated efficacy.

## Introduction

Kawasaki disease, an acute systemic vasculitis illness, causes CAA in about 25% of untreated patients; its occurrence rate could drop to 4% after treatment with intravenous immunoglobulins [1]. Meanwhile, 1% of cases develop giant CAA. In a small fraction of patients, acute coronary artery stenosis or obstruction occurs, even leading to acute myocardial infarction. Kawasaki disease has become one of the most common causes of pediatric acquired heart diseases in developing countries. Clinically, lesions can be classified into 5°; (1) I, normal coronary artery; (2) II, mild dilatation in the acute stage and recovery within 30 days; (3) III, one mild-to-moderate CAA; (4) IV, giant CAA or more than one aneurysm in one coronary artery without stenosis; (5) V, coronary angiography showing stenosis or obstruction, with or without myocardial ischemia. In pediatric patients with CAL of IV to V degrees or thrombosis, long-term therapy with low dose oral aspirin combined with warfarin or injection of low molecule-weight heparin is recommended [2].

Anticoagulant therapy has also been used in children for mechanical heart valve, Fontan operation, dilated cardiomyopathy, and all thrombosis types [3, 4]. Despite the discovery of new oral anticoagulants, including Xa factor- and thrombin-suppressants, Vitamin K antagonists (VKA), especially warfarin, remain the most common oral anticoagulants for long-term use. Warfarin has been used for more than half a century; however, due to its narrow therapeutic window, an irregular monitoring of laboratory outcomes, and poor patient compliance, the safety and efficacy of this active medicine still require further assessment. It is commonly admitted that warfarin diversity is considerable in different individuals, and can be partially explained by demographic, clinical, and environmental factors. Recently, genetic variations attract increasing attention, e.g. VKORC1, CYP2C9, CYP4F2, CYP1A2, CYP3A4, GGCX, APOE, and EPHX1 mutations. Among these genes, VKORC1, CYP2C9, and CYP4F2 have been studied more frequently.

This study aimed to determine non-genetic and genetic (CYP2C9, VKORC1, CYP4F2) factors affecting warfarin dose in Kawasaki disease. Warfarin is a racemic mixture of (*R*)-warfarin and (S)-warfarin.; (S)-warfarin, metabolized by CYP2C9, is three to five times more active as anticoagulant than (*R*)-warfarin [5, 6]. The most common mutation sites of CYP2C9 are *2 (SNP rs1799853) and *3 (SNP rs1057910), and activity of mutant CYP2C9 is reduced by 20–80%. VKORC1 is located on human chromosome 16p12-q21; it encodes the catalytic subunit of vitamin K epoxide reductase complex, which plays a key role in the vitamin K cycle. Warfarin plays a role of anticoagulation by suppressing VKORC1. CYP4F2 is also related to vitamin K metabolism and cycle [7]. In adults, CYP2C9 gene polymorphisms explain 5.7–27% of individual variations in warfarin dose studies, while VKORC1 and CYP4F2 mutations account for approximately 14.2–35% and 1.2–7%, respectively [8–11]. Currently, sex, age, height, weight, CYP2C9, and VKORC1 predict 50–60% warfarin diversity in total, with almost 40% of individual warfarin dose variations explained by genes affecting warfarin metabolism in adults. While the influences of CYP2C9, VKORC1, and CYP4F2 for warfarin dosage are not determinate in pediatric patients with Kawasaki disease.

## Materials and Methods

### Study Design

The enrolled pediatric patients, all from Southwest China, were recruited from the Children’s Hospital of Chongqing Medical University, in which they were hospitalized from October 2010 to July 2017. The children were composed of four groups. Cases with severe CAL of IV to V degrees or thrombogenesis treated with oral warfarin were assigned to Group A (experimental group). To compare mutation frequencies, 3 control groups were set up: Group B, CAL of I degrees; Group C, CAL of II and III degrees cases with small or medium-sized CAL not treated with warfarin; Group D, no Kawasaki disease (blank control). CAL degrees and warfarin indications were based on Recommendations for clinical management of Kawasaki disease with coronary arterial lesions [2]. CAA could be separated into three categories: (1) mild CAA, diameter ≤ 4 mm or diameter in older individuals (≥ 5 years) less than 1.5 times the normal value; (2) medium-sized CAA, diameter > 4 mm and ≤ 8 mm, or diameter in older individuals exceeding 1.5 to 4 times the normal value; (3) giant CAA, diameter > 8 mm or diameter in older individuals above 4 times the normal value. Echocardiography was used for coronary artery assessment.

### Sample Collection and Gene Sequencing

Venous blood (1.5 ml) from every patient was collected for DNA extraction with TINAamp Blood DNA Kit (TIANGEN). PCR was performed in a 25 µl volume containing 0.7 µM primer, 120 ng DNA, 2× Power Taq PCR MasterMix, and ddH2O; 35 cycles were performed to obtain the final products, which were sent to Beijing genomics institute (BGI) for CYP2C9 (*2 alleles, rs1799853;*3 alleles, rs1057910), VKORC1 (1173C > T, rs9934438), VKORC1 (− 1639G>A, rs9923231), VKORC1 (3730G>A, rs7294), and CYP4F2 (C > T, rs2108622) genotyping.

### Statistical Analysis

Data were presented as mean ± standard deviation (SD) and range for quantitative variables, and number and percentage for qualitative ones. Statistical analyses were performed with the SPSS Statistics Version 24.0 software. The Chi square test or Kruskal–Wallis test was used to compare gene mutation rates among the four groups. Pearson correlation analysis, unpaired *t* test, 2-specimen Wilcoxon test, and ANOVA were used to measure associations of warfarin dose with age, height, weight, body surface area (BSA), sex, target international normalized rate (INR), and various genotypes in univariate analysis. A stepwise multivariate regression model was performed to determine the predicted formula for warfarin dose. Parameters with *P* < 0.20 in univariate analysis were entered into multiple linear regression analysis. Two-sided *P* < 0.05 was considered statistically significant.

## Results

### General Features

A total of 59 children enrolled in Group A were hospitalized in the Children’s Hospital of Chongqing Medical University and assessed retrospectively; full data were obtained for 44 patients with a medium age of 3.6 years (3 months to 14.3 years), including 34 boys and 10 girls. Demographic and clinical characteristics, including target INR, cardiac thrombotic events, over-anticoagulation (defined as INR surpassing 4), warfarin dose, and others, are shown in Table 1. A total of 13 (29.6%) children had a target INR of 1.0–1.5, while 25 (56.8%) achieved a target INR of 1.5–2.0; the remaining had an INR of 2.0–2.5 (13.6%). Most patients had hyporrhea, including skin bruises, epistaxis, and bleedings gums; 2 children received reduced warfarin dosage because of frequent epistaxis, and 2 patients had severe bleeding episodes (gastrointestinal bleeding and traumatic intracranial hemorrhage, respectively) requiring emergency management. The occurrence rate of coronary artery thrombosis (CAT) was 75%, found once in 27 patients, twice in 4, and 3 times in 2. Meanwhile, 7 pediatric patients had over-anticoagulation, including 4 who were sick, 1 receiving traditional Chinese medicine, 1 using heparin simultaneously, and 1 increasing warfarin dosage without medical advice.

**Table 1:** Main characteristics of 44 patients in group A

	Mean ± SD (range) or N (%)
Age (m)	45.2 ± 41.7 (3–172)
Sex, male	34 (77.3)
Height (cm)	102.4 ± 24.3 (56–160)
Weight (kg)	17.8 ± 9.0 (6.5–53)
BSA (m^2^)	0.705 ± 0.267 (0.328–1.510)
Target INR	1.64 ± 0.34 (1.05–2.41)
Patients with thrombotic events	33 (75)
Over anticoagulation	7 (15.9)
Warfarin dose (mg/day)	1.705 ± 0.771 (0.4–3.5)
Warfarin dose (mg/kg/day)	0.101 ± 0.034 (0.036–0.222)
Follow-up time (m)	18.0 ± 13.0 (2.3–58.8)

Mean warfarin daily dosage was 0.101 ± 0.034 mg/kg (range 0.036–0.222 mg/kg), and was close to 0.1 mg/kg after grouping by age, weight, and target INR (Table 2). Older or heavier individuals required lower warfarin doses. Children with a target INR of 1.5–2 (1.728 ± 0.803 mg/day) had higher dosage compared with those with INR of 1.0–1.5 (1.689 ± 0.684 mg/day) and 2.0–2.5 (1.643 ± 0.941 mg/day).

**Table 2:** Warfarin dose by grouping in age, weight, and target INR

Dosage (mean ± SD) according to age group			
	Age < 2 years	2 years ≤ Age < 5 years	Age ≥ 5 years
mg/day	1.141 ± 0.459 (0.4–2.0)	1.504 ± 0.623 (0.5–2.75)	2.519 ± 0.505 (1.25–3.5)
mg/(kg day)	0.118 ± 0.043 (0.05–0.15)	0.095 ± 0.032 (0.036–0.120)	0.093 ± 0.022 (0.06–0.11)

Dosage (mean ± SD) according to weight group			
	Weight ≤ 10 kg	10 kg < weight ≤ 20 kg	Weight > 20 kg
mg/day	1.069 ± 0.458 (0.4–2)	1.442 ± 0.537 (0.5–2.625)	2.536 ± 0.489 (1.25–3.5)
mg/(kg day)	0.116 ± 0.046 (0.083–0.150)	0.098 ± 0.033 (0.036–0.156)	0.095 ±0.022 (0.047–0.120)

Dosage (mean ± SD) by difference target INR			
	1 < INR < 1.5	1.5 ≤ INR < 2	2 ≤ INR < 2.5
mg/day	1.689 ± 0.684 (0.833–2.75)	1.728 ± 0.803 (0.5–3.5)	1.643 ± 0.941 (0.4–2.625)
mg/(kg day)	0.102 ± 0.046 (0.047–0.222)	0.100 ± 0.027 (0.036–0.150)	0.102 ± 0.033 (0.05–0.146)

### Drugs and Foods

All children were administered other drugs besides warfarin, including aspirin (97.7%), fructose (29.5%), dipyridamole (15.9%), traditional Chinese medicine (11.4%), clopidogrel (6.8%), metoprolol (6.8%), levocarnitine (4.5%), benazepril (4.5%), tacrolimus (2.3%), vitamin E (2.3%), chalybeate (2.3%), calcium tablets (2.3%), and prednisone (2.3%). A total of 14/44 (31.8%) patients had special diets incorporating formula milk (n = 13, 29.5%; 1 infant took pure milk powder) and breastfeeding (n = 1, 2.3%). In our study, only clopidogrel and prednisone effected warfarin intake dose, and the effect was uncertain and cases involved were small.

### Genetic Findings

**Fig. 1:**
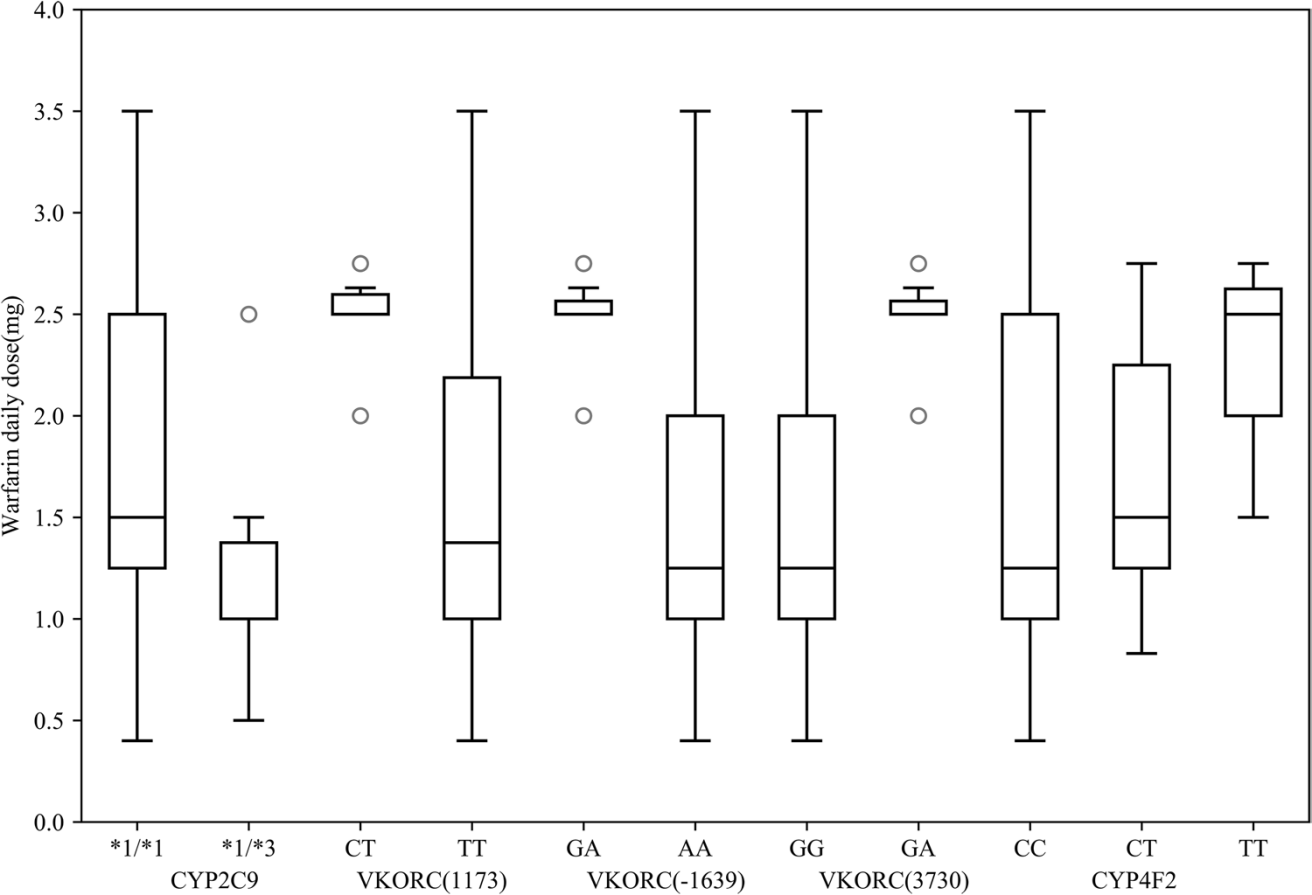
Box plots displaying warfarin daily dose by genotypes. It displayed the patients with wild-type homozygotes of CYP2C9 required more warfarin dose than that of heterozygotes, but patients with VKORC1 3730 and CYP4F2 presented the opposite results. And heterozygotes of VKORC1 (1173, − 1639) needed more dose than homozygotes, CYP2C9 genotype: *1/*1 wild-type homozygotes, *1/*3 heterozygotes; VKORC1 1173 genotype: CT heterozygotes, TT homozygotes; VKORC1 − 1639 genotype: GA heterozygotes, AA homozygotes; VKORC1 3730 genotype: GG wild-type homozygotes, GA heterozygotes; CYP4F2 genotype: CC wild-type homozygotes, CT heterozygotes; TT homozygotes

Children enrolled in Groups A to D were 59, 50, 35, and 50, respectively. The 4 groups showed no statistically significant differences in sex and age. Genetic findings are presented in Table 3. Gene mutation frequency of 4 groups showed no statistical difference. We combined group A, B, and C to compare with group D and the result was the same which reminded genotypes of CYP2C9, VKORC1 (1173, − 1639, and 3730), and CYP4F2 in different crowds were relatively stable. Of the 194 patients, no case had the CYP2C9*2 allele, while the CYP2C9*3 allele was rare (4.9%). The children had no wild-type genes, and mutant homozygotes were dominant for VKORC1 1173 and − 1639. VKORC1 3730 genotypes were only wild-type and heterozygotes; the mutation frequency of CYP4F2 TT in the 194 patients was less than 5%. Warfarin dosages in different genotypes are shown in Fig. 1.

**Table 3:** Comparison of genetic characteristics among 194 patients, *N* (%)

	A (*N* = 59)	B (*N* = 50)	C (*N* = 35)	D (*N* = 50)	Total (*N* = 194)	A (*N* = 44)
CYP2C9						
*1/*1	52 (88.1)	49 (98)	31 (88.6)	44 (88)	176 (90.7)	38 (86.4)
*1/*2	0	0	0	0	0	0
*1/*3	7 (11.9)	0	4 (11.4)	6 (12)	17 (8.8)	6 (13.6)
*2/*2	0	0	0	0	0	0
*2/*3	0	0	0	0	0	0
*3/*3	0	1 (2)	0	0	1 (0.5)	0
Wild allele	0.941	0.98	0.943	0.94	0.951	0.932
Variant allele	0.059	0.02	0.057	0.06	0.049	0.068
*2	0	0	0	0	0	0
*3	0.059	0.02	0.057	0.06	0.049	0.068
VKORC1						
1173:						
CC	0	0	0	0	0	0
CT	8 (13.6)	11 (22)	8 (22.9)	4 (8)	31 (16.0)	6 (13.6)
TT	51 (86.4)	39 (78)	27 (77.1)	46 (92)	163 (84.0)	38 (86.4)
Wild allele	0.068	0.11	0.114	0.04	0.08	0.068
Variant allele	0.932	0.89	0.886	0.96	0.92	0.932
− 1639						
GG	0	0	0	0	0	0
GA	9 (15.3)	11 (22)	8 (22.9)	4 (8)	32 (16.5)	7 (15.9)
AA	50 (84.7)	39 (78)	27 (77.1)	46 (92)	162 (83.5)	37 (84.1)
Wild allele	0.076	0.11	0.114	0.04	0.082	0.080
Variant allele	0.924	0.89	0.886	0.96	0.918	0.920
3730						
GG	50 (84.7)	39 (78)	27 (77.1)	46 (92)	162 (83.5)	37 (84.1)
GA	9 (15.3)	11 (22)	8 (22.9)	4 (8)	32 (16.5)	7 (15.9)
AA	0	0	0	0	0	0
Wild allele	0.924	0.89	0.886	0.96	0.918	0.920
Variant allele	0.076	0.11	0.114	0.04	0.082	0.080
CYP4F2						
CC	35 (59.3)	29 (58)	21 (60)	32 (64)	117 (60.3)	28 (63.6)
CT	20 (33.9)	21 (42)	13 (37.1)	15 (30)	69 (35.6)	13 (29.5)
TT	4 (6.8)	0	1 (2.9)	3 (6)	8 (4.1)	3 (6.8)
Wild allele	0.763	0.79	0.786	0.79	0.781	0.784
Variant allele	0.237	0.21	0.214	0.21	0.219	0.216

All pediatric patients in group A was 59, but only 44 cases were enrolled into the experimental group and total 194 people were detected genotypes of CYP2C9, VKORC1 (1173, − 1639, 3730), and CYP4F2

### Predicted Doses and Clinical Values

**Fig. 2:**
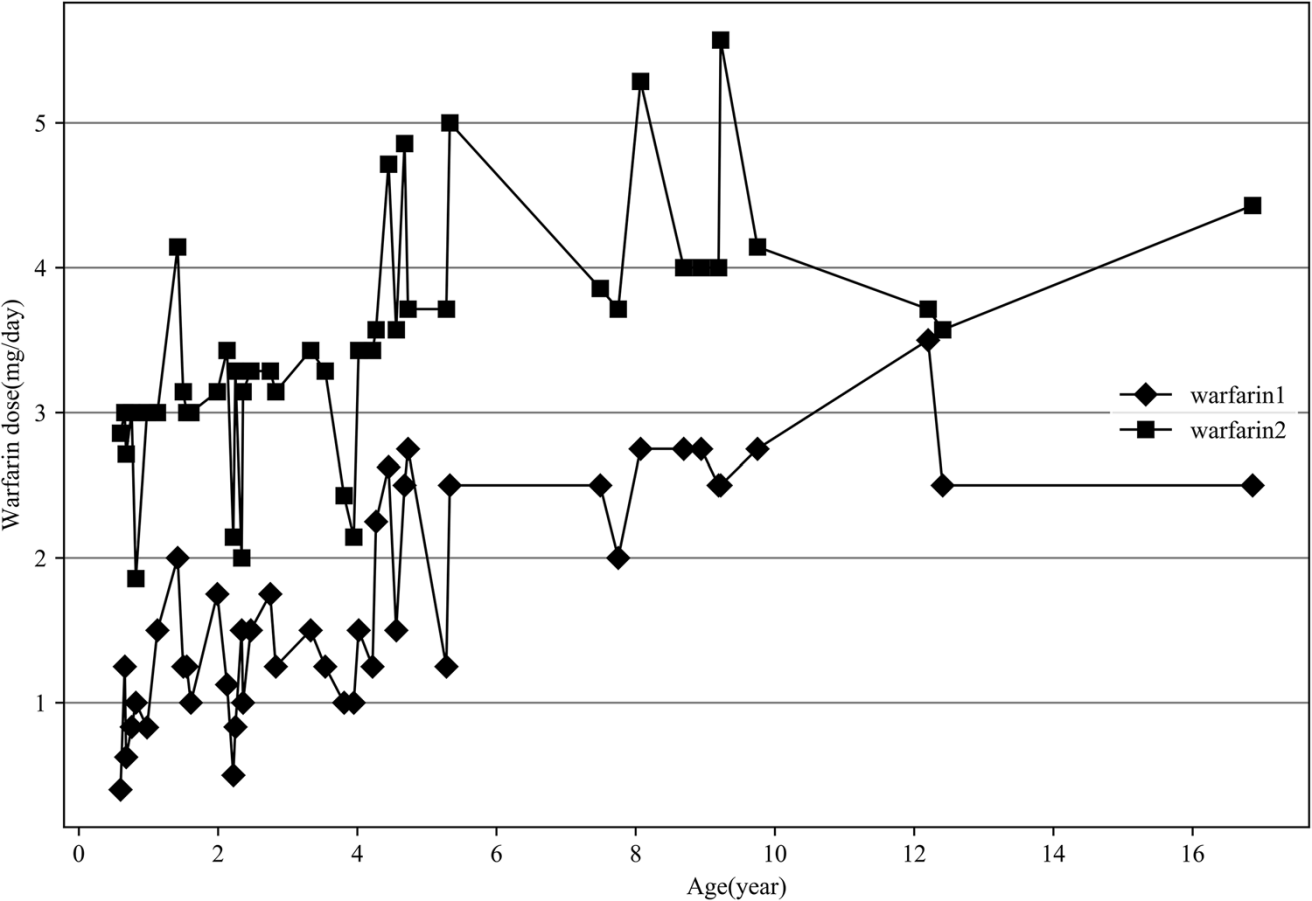
Relationship between age and actual daily warfarin dose or IWPC-predicted dose. It revealed that the prediction model of IWPC assessed more warfarin dose than actual dosage in group A. warfarin 1: actual daily warfarin dose, warfain 2: IWPC predicted dose

A specific quantitative formula was proposed by the International Warfarin Pharmacogenetics Consortium (IWPC), and included age, height, weight, race, CYP2C9 and VKORC1 − 1639, amiodarone, and enzyme inducers such as rifampin, phenytoin, and carbamazepine, for adult patients. Correction analysis showed a high relevance between the predicted warfarin daily dosage using the IWPC algorithm and clinical values (*r* = 0.742, *P* = 0.000). However, the prediction excessively estimated warfarin dose in 44 children (Fig. 2).

### Associations of Non-genetic and Genetic Factors with Warfarin Dose

#### Univariate Analysis

**Fig. 3:**
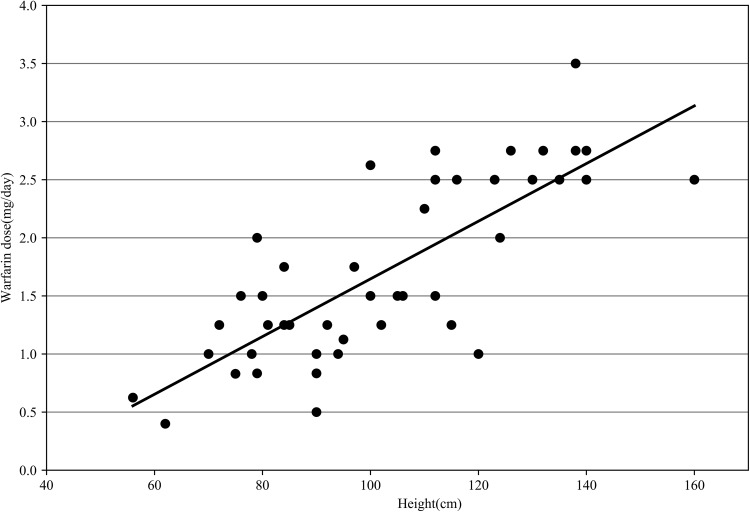
Relationship between height (cm) and warfarin dosage (mg/day). It indicated that warfarin dose were positive correlation with height

Warfarin daily dose was closely associated with age, height, weight, and BSA. Figure 3 shows the association of height with warfarin dosage. Univariate analysis revealed that sex (*P* = 0.056) and target INR (*P* = 0.968) were irrelevant to warfarin dose. The mean daily warfarin dose required in children with CYP2C9 *1/*1 (1.777 ± 0.767 mg; range 0.4–3.5 mg) was higher than that of individuals with CYP2C9 *1/*3 (1.250 ± 0.689 mg, range 0.5–2.5 mg, *P* = 0.121, − 29.7%), with 0.105 ± 0.033 mg/kg and 0.078 ± 0.032 mg/kg, respectively. Children with homozygous wild-type CYP4F2 (1.632 ± 0.833 mg) needed lower amounts than those with CT (1.737 ± 0.639 mg, *P* = 0.689, + 6.4%) and TT (2.250 ± 0.661 mg, *P* = 0.226, + 37.9%). Patients carrying one VKORC1 1173 variant allele (2.479 ± 0.255 mg) required relatively higher doses than TT homozygotes (1.583 ± 0.755 mg, *P* = 0.008, − 36.1%); children with VKORC1 − 1639GA (2.482 ± 0.233 mg) required a higher dose than those with AA (1.558 ± 0.749 mg, *P* = 0.004, − 37.2%), while VKORC1 3730 had the opposite result.

#### Multiple Regression

Age, height, weight, BSA, CYP2C9, VKORC1 1173, VKORC1 − 1639, and VKORC1 3730 were included in stepwise multiple linear regression analysis. Only height, CYP2C9, and VKORC1 1173 were significant (*P* < 0.001), and explained the large majority of individual variability in warfarin dose requirement (*R*^2^ = 73.4%; Table 4). Height, VKORC1 1173, and CYP2C9 accounted for 61.2%, 7.9%, and 4.3% variations, respectively.

**Table 4:** Multivariate regression model for predicting warfarin dose

Predictor	Coefficient	SE	*P*	Univariate *R*^2^
Height	0.023	0.003	0.000	0.612
VKORC1 1173	− 0.653	0.184	0.001	0.079
CYP2C9	− 0.460	0.182	0.015	0.043

Regression equation: dose (mg/day) = − 0.018 + 0.023 (height) − 0.653 (VKORC1 1173TT) − 0.46 (CYP2C9 *1/*3). Height: input centimeters; VKORC1 1173 genotype: input 0 for CT, 1 for TT; CYP2C9 genotype: input 0 for *1/*1, 1 for *1/*3, the other genotypes of VKORC1, and CYP2C9 didn’t be detected in 44 cases

The formula for predicting the dose was dose (mg/day) = − 0.018 + 0.023 × height (cm) − 0.653 × VKORC1 1173TT − 0.46 × CYP2C9 *1/*3.

## Discussion

Kawasaki disease, also called mucocutaneous lymph node syndrome, is an acute vasculitis disease in infants and young children. Inflammation in Kawasaki disease can cause wide polyangiitis, especially of the cardiovascular system, which can lead to coronary arteriectasis or even coronary aneurysm. Kawasaki disease with severe CAL or thrombotic formation requires a combination therapy of aspirin plus warfarin. Aspirin usage is fixed and valid, but warfarin dosage has large individual differences even in children with the same age or weight. Currently, a mature model for adults to predict warfarin dose is available, and studies increasingly focus on pediatric patients. Nevertheless, indications are different, and there might be distinct cardiovascular diseases, including the Fontan procedure, heart valve replacement, dilated cardiomyopathy, and coronary aneurysm, and other extra-cardiac diseases. No study has focused on only one disease such as Kawasaki disease. The primary reason might be fairly limited cases; we spent nearly 2 years of Southwest China, recruiting volunteers who matched inclusion criteria in the last 10 years, and only 44 patients were ultimately included in the experimental group. Another reason might be the viewpoint that indications possibly do not affect warfarin dose. In warfarin therapy for Kawasaki disease, clinicians focus more on dose-adjustment depending on clinical experience. Our research team comprises experts in the cardiovascular field, with great interest in warfarin treatment of Kawasaki disease, which is the most common acquired heart disease in developing countries. Besides, previous studies generally do not set control groups when assessing associations of warfarin dose and genotypes. Peng et al. [12] demonstrated that 4 gene loci, including FCGR2A rs1801274, BLK rs2254546, CD40 rs4813003, and HLA rs2857151, are associated with Kawasaki disease. ITPKC and CASP3 were linked with CAL in Kawasaki disease [13]. Control groups are necessary to provide more comprehensive findings.

This study assessed the associations of warfarin dose with genetic and non-genetic variables in children with Kawasaki disease, with appropriate control groups in order to compare genotype mutations of CYP2C9, VKORC1, and CYP4F2. Most children in the warfarin-treatment group were 6 months to 5 years, which is the common age of patients with Kawasaki disease. Height, VKORC1 1173, and CYP2C9 determined warfarin dose, and accounted for 73.4% of warfarin dose variability; patient height accounted for 61.2%, representing the main contributor. There were no statistically significant differences in CYP2C9, VKORC1, and CYP4F2 among the four groups, not supporting the implication of these genes in Kawasaki disease, CAL occurrence, or CAL severity.

The first prospective cohort study with 59 patients the associations of VKORC1, CYP2C9, and VKA dosage in children demonstrated that age, VKORC1, and CYP2C9 explained 38% of dose variations, with age constituting the most important factor (28.3%) [14]. However, anticoagulants included not only warfarin but also phenprocoumon, and most patients received steroids, which could affect the curative effect. In this study, age was also significantly associated with warfarin dosage in univariate analysis; however, was not significant in multivariate analysis. Indeed, many studies hold the view that age is strongly associated with height and weight. Assessing 120 children from four United Kingdom sites, Tina et al. [8] also demonstrated that height is a superior dose predictor compared to age or weight, which may be due to the close relationship between height and liver size. Higher doses of anticoagulants and vitamin K in heparin are likely used with increasing height, and this study revealed that height was a more representative clinical index than the other factors.

In the final model, CYP2C9 and VKORC1 1173 genotypes explained 4.3% and 7.9% interindividual variability of warfarin dose, respectively. These proportions were relatively lower than those reported in most adult and pediatric studies. CYP2C9 variant allele carriers (*2 or *3) require lower warfarin dosages than individuals with wild type allele (*1). In the present cohort, CYP2C9*1/*3 carriers required significantly lower warfarin doses than individuals with CYP2C9*1/*1; meanwhile, mutant genotypes were relatively rare, and the effects of other mutant CYP2C9 genotypes could not be evaluated. Adult patients with CYP2C9 mutations have high rates of serious or life-threatening bleeding and above-INR compared with wild-type counterparts [15]. Kaitlyn et al. [9] reported that CYP2C9*3 carriers show higher risk of major bleeding, while those with variant VKORC1 alleles have a trend of bleeding. Similar results were obtained in this study. Taken together, these findings revealed increased risk of bleeding in CYP2C9 and VKORC1 variant allele carriers.

In Group A, no patient had the wild-type VKORC1 1173 or − 1639 gene, but showed homozygous mutations (1173TT, 84%; − 1639AA, 83.5%); the mutation rate was higher than those reported for white and African American individuals, and similar to that of Asians. The patients with VKORC1 1173 CC required higher warfarin doses compared with the CT or TT groups. There was no wild type individuals, but the trend was consistent; compared with heterozygotes, cases with homozygous mutations required 36.1% less warfarin. Studies indicated that patients with VKORC1 − 1639 AA require significantly lower warfarin doses than the GG or GA groups. Avisa et al. [5] reported opposite results, with only two patients with the AA genotype not receiving higher doses. Studies indicated that 1173 and − 1639 SNP are in strong linkage disequilibrium (LD), and even the three SNPs VKORC1 (1173, − 1639, and 3730) might be in LD. Such conclusions do not apply to other races but individuals with Asian-descent. We found that 1173C > T mutation was not entirely consistent with − 1639G>A, since one of the 194 patients had inconsistent results. While VKORC1 1173 and VKORC1 − 1639 were not in strict LD, conclusions regarding warfarin dose were highly similar; VKORC − 1639 and VKORC 3730 were seemingly in LD. These findings indicated it was unnecessary to take all these genes into consideration simultaneously. CYP4F2 mutations showed results similar to those previously reported. Children carrying a variant allele of CYP4F2 required higher doses than wild-type counterparts (CT, 6.4%; TT, 37.9%), and we found no associations of CYP4F2 genotypes with warfarin dosage in the prediction model with a finite number of cases.

Medicines and food could also affect warfarin dose. Children on phenobarbital/carbamazepine or receiving enteral nutrition required elevated doses [16]. Drugs and diet types potentiate or attenuate warfarin effects [2, 13]. It was even proposed that the dietary state of vitamin K possibly explains most individual differences besides clinical and genetic factors. Assessing drug effects, Kaitlyn et al. [9] analyzed over 10 concomitant drugs, and found no associations with warfarin dose. To evaluate the effects of food, we compared warfarin dosages between children partly receiving milk powder and those with normal diet, and found the former required lower doses than the latter (1.203 ± 0.439 mg vs. 1.957 ± 0.769 mg, *P* = 0.004); however, the former cases were all infants. A study of 300 adults showed vegetarian or accidentally nonvegetarian patients intake lower warfarin doses than non-vegetarians [17]. Besides, diverse warfarin indications require different daily doses. Kaitlyn et al. [9] found the Fontan procedure demanded a lower dose (2.5 ± 1.2 mg) than deep vein thrombosis/pulmonary embolism patients (5.0 ± 2.6 mg; *P* = 0.002) or other diseases (4.7 ± 2.4 mg; *P* = 0.001). This may be related to increased sensitivity, abnormal liver function, loss of appetite, reduced intake of vitamin K, and lack of exercise after surgery [18, 19].Interestingly, ancestry affects warfarin dosage, with Indian and Pakistani patients requiring higher doses compared with other ethnic origins [8]. The current cohort included only Kawasaki disease patients from Southwest China, avoiding the effects of indications and ethnicity. Studies assessing drugs, food, indications, and races are relatively rare, partly because they are very hard to design and execute; in addition, their potential effects on warfarin dose are not well-defined.

A limitation of this study was its type, i.e., a single institution retrospective study, which failed to control for or/and analyze some factors, including test frequency of echocardiography and INR, the specific doses of other drugs, and diets. Another shortcoming was sample size in the experimental group, which resulted in reduced number of VKORC1 genotypes.

## Conclusions

This study demonstrated that height and VKORC1 1173 and CYP2C9 genotypes significantly determine warfarin dose in Kawasaki disease in Southwest Chinese. Warfarin dosage models based on pharmacogenetics have been generated for adults, but are not used in pediatric patients due to excessive estimation of warfarin dosage. Meanwhile, studies have explored the efficiency of warfarin dose based on genotyping [5, 20, 21], but most of them had small sample sizes and unclear findings. To further define warfarin pharmacogenetics in children, multi-center, randomized control trials are warranted.
